# The Star Recurrent Vaginal Syringe Point

**Published:** 1889-04

**Authors:** 


### THE STAR RECURRENT VAGINAL SYRINGE POINT.

This is a useful apparatus for administering vaginal injections in all pelvic diseases where copious lavements, plain or medicated, are required. It is also adapted to washing out the vagina in obstetric practice, both before and after labor. It is so constructed as to prevent soiling or wetting the clothing or bed-linen, rendering the use of the bed-pan or other receptacle for the waste fluid unnecessary. It is readily adapted to any bulb-syringe, or can be used with a fountain attachment. Manufactured and for sale by the Star Rubber Company, Lafayette, Ind.

